# Community medical service construction: identifying factors that influence medical choice for patients with non-communicable chronic diseases in the Southwest China

**DOI:** 10.1186/s12889-024-18789-z

**Published:** 2024-05-20

**Authors:** Xue Zhang, Jing Dai, Wei Li, Yu Chen, Yunyu He, Yunjuan Yang, Liuyang Yang

**Affiliations:** 1 Faculty of Management and Economics, Kunming University of Science and Technology, Kunming, 650093 PR China; 2 Sheng Ai Hospital of Traditional Chinese Medicine, Kunming, 650051 PR China; 3 Party Committee Office, Kunming Medical University, Kunming, 650051 PR China; 4 The First People’s Hospital of Yunnan Province, Kunming, 650034 PR China; 5 Yunnan Provincial Center for Disease Control and Prevention, Kunming, 650022 PR China; 6 School of Population Medicine and Public Health, Chinese Academy of Medical Sciences & Peking Union Medical College, Beijing, 100730 PR China

**Keywords:** Community medical service, Patients with NCDs, Medical choice, Southwest underdeveloped areas

## Abstract

**Background:**

Community medical institutions play a vital role in China’s healthcare system. While the number of these institutions has increased in recent years, their construction contents remain insufficient. The potential of community medical institutions in preventing, screening, diagnosing, and treating non-communicable chronic diseases (NCDs) has not been fully utilized. This study aims to assess the status of construction contents in community medical institutions in Southwest China and examine how these contents influence the medical choices of NCD patients.

**Methods:**

Descriptive statistics were used to evaluate the construction content of community medical institutions. Multiple-sets of multinomial logistic regression were employed to analyze the associations and marginal impacts between construction content and medical choices. Shapley value analysis was applied to determine the contribution and ranking of these impacts.

**Results:**

Descriptive statistics revealed satisfactory construction contents in community medical institutions. Notably, factors such as service attitude, nursing services, expert consultations, charging standards, medical equipment, medical examinations, privacy protection, and referrals significantly influenced medical choices. Among these, service attitude, charging standards, and privacy protection had the most significant marginal improvement effects on NCD patients’ choices, with improvements of 12.7%, 10.2%, and 5.9%, respectively. The combined contribution of privacy protection, medical examinations, service attitude, charging standards, and nursing services to medical choices exceeded 80%.

**Conclusion:**

Optimizing the service contents of community institutions can encourage NCD patients to seek medical care at grassroots hospitals. This study addresses crucial gaps in existing literature and offers practical insights for implementing new medical reform policies, particularly in underdeveloped regions of Southwest China focusing on hierarchical diagnosis and treatment.

## Introduction

Non-communicable chronic diseases (NCDs) refer to a class of disease that exist for a long time and are difficult to cure [1], such as hypertension, diabetes, stroke, etc. Patients with NCDs need long-term treatment and even lifelong medication. Especially, the prevalence of NCDs is rising with the rapidly aging population in China [2]. Unlike most western countries that have had this transition at a slower pace, China has experienced this shift only in a few decades [3, 4], which has consequently caused a rapid increase in NCDs burden in China. Moreover, many patients with NCDs prefer to choose provincial and district hospitals for diagnosis and treatment [5]. This not only takes up the precious medical resources of the tertiary hospitals but also increases the family medical expenses, reduces the convenience of medical treatment. Hence, selecting community medical institutions can not only reduce medical expenses, but also reduce queuing time and improve medical experience, for patients with NCDs and who need routine follow-up. Conversely, the current situation of medical treatment for Chinese is that more than 54% of patients choose tertiary hospitals, including patients with mild chronic diseases and in regular follow-up [6]. It has led to a series of problems such as the over-burden of tertiary hospitals, the idle medical resources of community medical institutions, and the poor experience of patients. To date, much of the research on medical choice of patients with NCDs focuses on the medical choice behavior, influencing factors of medical choice behavior, and medical reform policies such as hierarchical diagnosis and treatment, medical union influencing the behavior of medical choice [7–9]. While important, those studies only focus on the medical behavior of patients with NCDs and their macro influencing factors, which is to change the medical behavior of NCDs patients from passive side. Previous research indicates that the poor service quality of community medical institutions is an important factor affecting the choice of patients with NCDs [10]. Nevertheless, the impact of each construction content of community medical institutions on the choice of treatment for patients with NCDs is little known.

Since 2009, China has issued a series of medical reform policies to promote the construction of primary medical institutions. Chinese “new medical reform” takes improving the primary medical services as one of the goals [11]. On the one hand, the government allocated more funds to primary medical institutions. On the other hand, China has also implemented a series of “push” strategies to lead patients to community medical institutions. It includes compulsory first visit of residents in the community, differentiated medical insurance reimbursement [12], and implementation of family doctor contract system [13]. Moreover, “pull” strategies such as improving medical quality, medical facilities, professionals, and other aspects are also needed.

However, the imbalance of medical resources allocation between regions, between urban and rural areas, and between different levels of medical institutions has been prominent in China for a long time [14–16]. The allocation of medical and health resources in China shows a trend that the east is better than the west, the city is better than the countryside, and the provincial hospital is better than the community hospital. Southwest is an underdeveloped region in China [17]. Its level of urbanization and economic development is lower than that of the eastern coastal areas, and the medical resources are also relatively insufficient. Particularly, the medical and health resources of community hospitals in southwest China are seriously insufficient. It is common for NCDs patients to delay seeking medical treatment due to the high cost of chronic disease diagnosis and treatment in hospitals. Previous studies have shown that the two-week visit rate for NCDs patients in the western region is 12.0% [6], which is 1.0% lower than the two-week visit rate for the general population nationwide. Hence, how to make limited medical resources benefit more patients and better serve them? This is the key to the “pull” strategy and solving the problem of difficult and expensive medical treatment for residents.

In the context of high prevalence of NCDs and construction of community medical institutions, the purpose of the current study is to examine the following three questions.

(1) What is the current situation of each construction content of the community medical institutions and the medical choice of NCDs patients?

(2) If there any associations between construction content of the community medical institutions and the medical choice of NCDs patients?

(3) What are the marginal impact and contribution of each construction content of community medical institutions on the medical choice of NCDs patients?

## Materials and methods

### Data collection

**Fig. 1:**
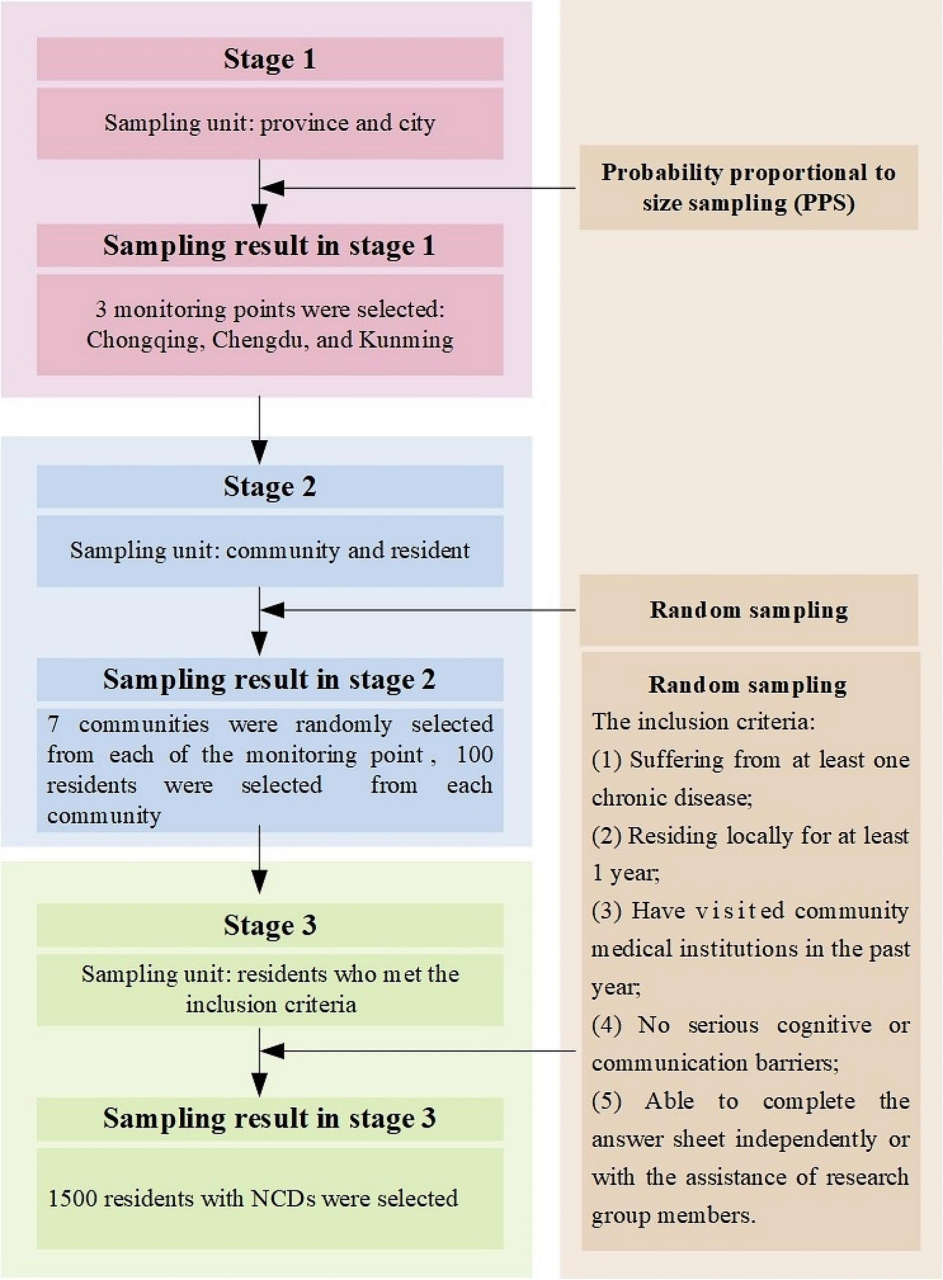
Sampling process

This research was a cross-sectional study, which was carried out in the Southwest areas of China from December 2021 to December 2022. The subjects were selected by stratified cluster random sampling method. Fig. 1 shows the sampling procedure. In the first stage, 3 monitoring points were selected by probability proportional to size sampling (PPS) from 64 regions in Southwest China. The three monitoring points were Chongqing, Chengdu, and Kunming respectively. These three cities were with the highest level of economic development and medical resource in the Southwest China. The amounts of provincial hospitals in these three cities were 61, 91, and 34, respectively. And the premature mortality rates of major NCDs residents in the three cities were 13.66%, 16.08%, and 11.45% by the 2022 [18]. In the second stage, 7 communities were randomly selected from each of the monitoring point. In the third stage, 100 residents from each community were selected by random sampling method. Residents with NCDs who met the inclusion criteria were recruited from the selected communities for a questionnaire survey. The inclusion criteria were, (1) Suffering from at least one chronic disease; (2) Residing locally for at least 1 year; (3) Have visited community medical institutions in the past year; (4) No serious cognitive or communication barriers; (5) Able to complete the answer sheet independently or with the assistance of research group members.

The project team was composed of the staff of the First People’s Hospital of Yunnan Province, Yunnan Provincial Center for Disease Control and Prevention, and the teachers and graduate students of Kunming University of Science and Technology. The members of the project team had passed the unified and standardized pre-investigation training. To avoid the threat of demand characteristics in surveys, the project implementation principles are as follows. Firstly, the responses of participants were anonymous and would be used for research purposes only. Secondly, the project members were asked to use neutral and non-leading language to prevent bias and cues that may influence participants’ response. Thirdly, pilot testing was done before the formal survey to identify and address any potential demand characteristics or ambiguities. Subsequently, all participants provided their written consent. This study was approved by the Ethics Review Committee of the First People’s Hospital of Yunnan Province (approval number 2022ZYFB001).

In the end, a total of 1500 questionnaires were distributed and 1238 questionnaires were collected. After excluding 33 invalid questionnaires (such as incomplete and logically incorrect questionnaires), 1205 questionnaires were obtained, with an effective recovery rate of 80.33%.

### Measurement

#### Outcome variable (medical choice)

Participants were asked “What is the most serious chronic health problem that bothers you at present?” If the respondents choose any of the 14 NCDs such as hypertension, diabetes, stroke, etc. in the list, then they would continue to be asked “What type of medical institutions do you mainly visit in the past year?” the answer included “provincial hospitals”, “district hospitals”, and “community hospitals”. In this study, provincial hospitals refer to tertiary medical institutions that can provide high-level specialized medical services to multiple regions and carry out higher education and research tasks. They can provide high-level diagnostic and treatment services for NCDs patients, while also promoting innovation and improvement in NCDs management. District hospitals refer to secondary medical institutions that can provide comprehensive medical and health services to multiple communities and undertake certain teaching and research tasks. They play a bridging role in the management of NCDs. They can handle both complex NCDs cases and undertake referral tasks for NCDs patients that grassroots hospitals cannot handle. Community hospitals refer to grassroots medical institutions, such as township health centers, village clinics, community medical institutions, etc., which directly provide prevention, health care, and rehabilitation services to a certain population of communities. Community hospitals play the role of gatekeepers in NCDs management, mainly responsible for the prevention, screening, initial diagnosis and treatment of NCDs, as well as long-term follow-up of patients.

#### Exposure variables (construction content of community medical institutions)

According to previous research about quality assessment of community medical institutions and effect evaluation of community chronic disease management [19, 20], the construction content of community medical institutions is divided into four categories, including medical and nursing quality, cost of diagnosis and treatment, hardware facilities, and external factors. Medical and nursing quality included four positive indicators: service attitude, nursing service, doctor-patient communication, and expert consultation. Participants were promoted with questions, “How would you rate the service attitude/ nursing service/ doctor-patient communication of your community medical institution?” The values on the scale from one to five respectively corresponded to being “Very bad”, “Bad”, “General”, “Good”, and “Very good”. In addition, the question, “How often would your community medical institution offer expert consultation services?” was also queried. The answer scale from one to five was “Never”, “Occasionally”, “Sometimes”, “Often”, and “Always”.

Waiting time and charging standard variables were included in cost of diagnosis and treatment, in which the former was a negative factor and the latter was a positive factor. Participants were asked, “How long would waiting in the community medical institution?” The answers included 1 “Within half an hour”, 2 “Half an hour to one hour”, 3 “One hour to two hours”. In addition, the question “How would you rate the charging standard of your community medical institution?” was also quired. The answers included 1 “Very unreasonable”, 2 “Unreasonable”, 3 “General”, 4 “Reasonable”, 5 “Very reasonable”.

Hardware facilities included one negative indicator and two positive indicators, which were drug type, medical equipment, and medical examination respectively. The query posed was, “Please assess whether the variety of drugs in your community medical institution satisfy your requirement?” The response included 1 “satisfied”, 2 “general”, 3 “unsatisfied”. What’s more, another question was also asked, “How would you rate the progressiveness of medical equipment in your community medical institution?” The answers included 1 “Very not advanced”, 2 “Not advanced”, 3 “General”, 4 “Advanced”, 5 “Very advanced”.

External factors included three positive variables: distance to visit, privacy protection, and referral. Participants were promoted with questions, “How would you rate the distance to visit/ privacy protection/ referral in your community medical institution?” The values on the scale from one to five respectively corresponded to being “Very bad”, “Bad”, “General”, “Good”, and “Very good”.

### Statistical analysis

The current study used descriptive statistics, multiple sets of multinomial logistic regression analysis, and shapley value decomposition method to examine three research questions. First, to determine the current situation of each construction content of community medical institutions and the medical choice of NCDs patients with different characteristics (Research Question #1). Next, to explore the associations between each content of community medical institutions and medical choice of patients with NCDs (Research Question #2), each construction content of community medical institutions was entered into Model l, without controlled the demographic information and historical health information that was significant in the Research Question #1. Then, the demographic information and historical health information was controlled step by step, enter into Model 2 and Model 3 respectively. Thirdly, to further identify the marginal impact and contribution of each construction content of community medical institutions on the medical choice of NCDs patients (Research Question #3), the shapley value decomposition method and further calculation of multinomial logistic regression analysis were applied.

## Results

### Descriptive analysis for the sample

**Fig. 2:**
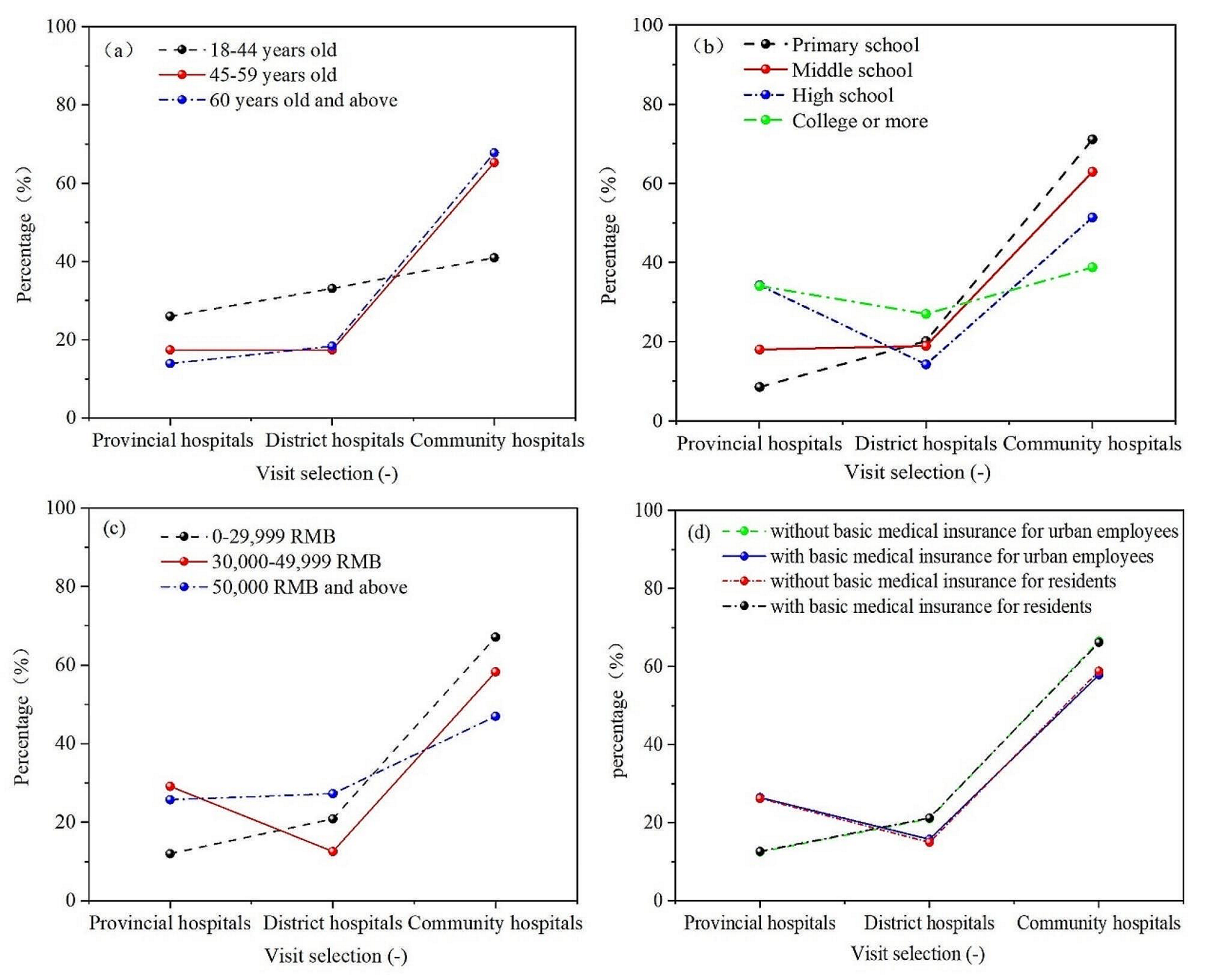
Medical choice of NCDs patients with different characteristics

Table 1 showed baseline characteristics of patients with NCDs who chose provincial, district, and community hospitals to visit. Compared with provincial and district hospitals, patients with NCDs preferred to choose community hospitals (64.40% vs. 19.93%, 19.67%). Age groups, education attainment, individual income, basic medical insurance for urban employees, and basic medical insurance for residents had a significant impact on medical choice for patients with NCDs (*p* < 0.001). The specific impact could be seen in Fig. 2. Compared to patients with NCDs who were under 45 years old, those 45 years old and above were more likely to choose community hospitals (65.28% vs. 40.94%). With the improvement of education, the proportion of patients with NCDs choosing community hospitals had declined. The percentage of NCDs patients with primary school education choosing community hospitals was 71.20%, while the percentage of NCDs patients with college or more education choosing community hospitals was 38.82%. The choice of hospitals for patients with NCDs was directly proportional to the individual income. The higher the individual income, the higher the level of medical choice. The probability of NCDs patients with individual income below 30,000 yuan and those with individual income above 50,000 yuan choosing community hospitals was 67.14% and 46.97% respectively. Additionally, those NCDs patients with basic medical insurance for residents were more likely to choose community hospitals compared with those with basic medical insurance for urban employee (66.59% vs. 57.86%).

**Table 1:** Baseline variables by groups depending on medical choice

Item	Total *N* (%)	Provincial hospitals *N* (%)	District hospitals *N* (%)	Community hospitals *N* (%)	*χ* ^*2*^	*P* value
Total	1205(100.00)	192(15.93)	237(19.67)	776(64.40)	-	-
**Gender**					3.597	^ns^
Male	476(39.50)	87(18.28)	87(18.28)	302(63.44)		
Female	729(60.50)	105(14.40)	150(20.58)	474(65.02)		
**Age groups (years)**					35.881	^***^
Q1 (18–44)	127(10.54)	33(25.98)	42(33.07)	52(40.94)		
Q2 (45–59)	265(21.99)	46(17.36)	46(17.36)	173(65.28)		
Q3 (≥ 60)	813(67.46)	113(13.90)	149(18.33)	551(67.77)		
**Educational attainment**					92.032	^***^
Primary school	653(54.19)	56(8.58)	132(20.21)	465(71.21)		
Middle school	327(27.14)	59(18.04)	62(18.96)	206(63.00)		
High school	140(11.62)	48(34.29)	20(14.29)	72(51.42)		
College or more	85(7.05)	29(34.12)	23(27.06)	33(38.82)		
**Marital status**					0.874	^ns^
Married	993(82.41)	156(15.71)	200(20.14)	637(64.15)		
Unmarried/divorced/widowed	212(17.59)	36(16.98)	37(17.45)	139(65.57)		
**Individual income (RMB/ Year)**					51.092	^***^
Q1 (0–29,999)	916(76.02)	110(12.01)	191(20.85)	615(67.14)		
Q2 (30,000–49,999)	223(18.51)	65(29.15)	28(12.56)	130(58.29)		
Q3 (≥ 50,000)	66(5.47)	17(25.76)	18(27.27)	31(46.97)		
**Basic medical insurance for urban employees**					33.246	^***^
No	906(75.19)	113(12.47)	190(20.97)	603(66.56)		
Yes	299(24.81)	79(26.42)	47(15.72)	173(57.86)		
**Basic medical insurance for residents**					35.002	^***^
No	316(25.23)	83(26.27)	49(15.51)	184(58.22)		
Yes	889(74.77)	109(12.26)	188(21.15)	592(66.59)		
**Commercial medical insurance**					1.497	^ns^
No	1198(99.42)	190(15.86)	235(19.62)	773(64.52)		
Yes	7(0.58)	2(28.57)	2(28.57)	3(32.86)		

^*^ significant at 5%, ^**^ significant at 1%, ^***^ significant at 0.1%, ^ns^ not significant

### Current situation of each construction content of community medical institutions

As shown in Table 2, most of the patients with NCDs rated the content construction of community hospitals at more than 4 points (the mean value over 4), and the patients with NCDs rated the service attitude and privacy protection of community hospitals the highest, with the mean of 4.43 and 4.42 respectively. Notably, the construction of medical equipment and drug type was a shortage, with 3.92 and 1.60 average score respectively.

**Table 2:** Definition and descriptive statistics of construction content of community medical institution

Construction content	Variable definition	Mean	*SD*
Service attitude	Very bad = 1, bad = 2, general = 3, good = 4, very good = 5	4.43	0.87
Nursing service	Very bad = 1, bad = 2, general = 3, good = 4, very good = 5	4.39	0.83
Doctor-patient communication	Very bad = 1, bad = 2, general = 3, good = 4, very good = 5	4.33	0.79
Expert consultation	Never = 1, occasionally = 2, sometimes = 3, often = 4, always = 5	4.24	0.77
Waiting time	Within half an hour = 1, half an hour to one hour = 2, one hour to two hours = 3	1.24	0.46
Charging standard	Very unreasonable = 1, unreasonable = 2, general = 3, reasonable = 4, very reasonable = 5	4.25	0.81
Drug type	Satisfied = 1, general = 2, unsatisfied = 3	1.60	0.61
Medical equipment	Very not advanced = 1, not advanced = 2, general = 3, advanced = 4, very advanced = 5	3.92	0.97
Medical examination	Very not helpful = 1, not helpful = 2, general = 3, helpful = 4, very helpful = 5	4.16	0.83
Visit distance	Very inconvenient = 1, inconvenient = 2, general = 3, convenient = 4, very convenient = 5	4.37	0.79
Privacy protection	Very bad = 1, bad = 2, general = 3, good = 4, very good = 5	4.42	0.81
Referral	Very bad = 1, bad = 2, general = 3, good = 4, very good = 5	4.20	0.83

### Association between content of community medical service and medical choice

As shown in Table 3, most of the construction contents of community medical institutions had significant impact on the medical choice of patients with NCDs. Compared to provincial hospitals, the construction content of community medical institutions had a greater effect on district hospitals. In other words, improving construction content of community medical institutions was more conducive to promoting the flow of NCDs patients in district hospitals to community hospitals. When demographic and historical health information were not controlled, service attitude, nursing service, expert consultation, charging standard, medical equipment, medical examination, privacy protection, and referral had a significant impact on NCDs patients from district hospitals to community hospitals. When demographic and historical information were gradually controlled, other construction contents still had significant effect on medical choice of NCDs patients besides referral. Additionally, only the construction of medical equipment, medical examination, and privacy protection in the community would promote NCDs patients who chose provincial hospitals to choose community hospitals. And with the control of demographic and historical health information, its significance remained unchanged.

**Table 3:** The impact of construction content of community medical institution on medical choice of patients with NCDs

Item	Model 1		Model 2		Model 3	
	Provincial VS community hospitals	District VS community hospital	Provincial VS community hospitals	District VS community hospital	Provincial VS community hospitals	District VS community hospital
Service attitude	0.007 [-0.382, 0.397]	-0.789^***^ [-1.137, -0.442]	0.223 [-0.182, 0.628]	-0.877^***^ [-1.233, -0.521]	0.205 [-0.208, 0.618]	-0.916^***^ [-1.278, -0.554]
Nursing service	0.284 [-0.125, 0.693]	-0.430^**^ [-0.768, -0.093]	0.141 [-0.272, 0.554]	-0.463^**^ [-0.812, -0.113]	0.196 [-0.230, 0.619]	-0.477^**^ [-0.822, -0.132]
Doctor-patient communication	0.049 [-0.312, 0.411]	0.019 [-0.305, 0.344]	0.086 [-0.298, 0.470]	0.005 [-0.323, 0.334]	0.056 [-0.337, 0.451]	-0.137 [-0.344, 0.317]
Expert consultation	0.098 [-0.190, 0.385]	-0.483^*^ [-0.598, -0.037]	0.023 [-0.270, 0.317]	-0.280^*^ [-0.530, -0.030]	0.035 [-0.298, 0.299]	-0.300^*^ [-0.553, -0.047]
Waiting time	0.091 [-0.274,0.455]	0.043 [-0.298, 0.384]	-0.027 [-0.408, 0.355]	-0.046 [-0.397, 0.304]	-0.048 [-0.442, 0.344]	-0.063 [-0.423, 0.297]
Charging standard	-0.203 [-0.485, 0.079]	-0.684^***^ [-0.934, -0.433]	-0.253 [-0.553, 0.046]	-0.662^***^ [-0.916, -0.408]	-0.248 [-0.553,0.058]	-0.694^***^ [-0.954, -0.435]
Drug type	-0.165 [-0.448,0.117]	-0.104 [-0.364, 0.156]	-0.224 [-0.519, 0.072]	-0.168 [-0.432, 0.097]	-0.223 [-0.526, 0.081]	-0.168 [-0.436, 0.100]
Medical equipment	-0.202^*^ [-0.385, -0.018]	-0.466^**^ [-0.797, -0.233]	-0.296^**^ [-0.494, -0.097]	-0.457^**^ [-0.756, -0.153]	-0.306^**^ [-0.512, -0.101]	-0.411^*^ [-0.632, -0.323]
Medical examination	-0.216^**^ [-0.486, -0.054]	-0.324^*^ [-0.712, -0.131]	0.198 [-0.086, 0.482]	-0.298^**^ [-0.619, -0.072]	-0.228^**^ [-0.519, -0.063]	-0.193^*^ [-0.355, -0.068]
Visit distance	-0.198 [-0.516, 0.119]	-0.128 [-0.400, 0.145]	-0.318 [-0.647, 0.011]	-0.155 [-0.432, 0.122]	-0.326 [-0.660, 0.008]	-0.142 [-0.421, -0.137]
Privacy protection	-0.520^**^ [-0.846, -0.194]	-0.292^*^ [-0.555, -0.029]	-0.624^***^ [-0.972, -0.276]	-0.340^*^ [-0.608, -0.072]	-0.582^**^ [-0.933, -0.230]	-0.322^*^ [-0.593, -0.052]
Referral	0.157 [-0.263, 0.295]	-0.168^**^ [-0.352, -0.071]	0.097 [-0.200, 0.395]	-0.018 [-0.279,0.242]	0.132 [-0.172, 0.435]	0.015 [-0.263, 0.266]
Demographic information	Uncontrolled	Uncontrolled	Controlled	Controlled	Controlled	Controlled
Historical health information	Uncontrolled	Uncontrolled	Uncontrolled	Uncontrolled	Controlled	Controlled
Prob > *chi*^*2*^	0.161		0.233		0.251	
Pseudo *R*^*2*^	0.000		0.000		0.000	

### Effects and contribution of the construction content in community medical institution on medical choice

It could be seen from Table 4 that the construction content of community medical institutions had significant marginal impacts on the medical choice of patients with NCDs. The service attitude construction of community medical institutions had reduced the probability of NCDs patients choosing district hospitals by 12.4%, while increased the probability to community hospitals by 12.7%. In addition, constructing nursing service in the community medical service had reduced the probability of NCDs patients choosing distinct hospitals by 7.3%, while enhanced the probability to community hospitals by 3.6%. The expert consultation for the construction of community medical institutions reduced the probability for patients with NCDs choosing district hospitals by 4.4%, while increased the probability of choosing community hospitals by 3.5%. Moreover, the standardized construction of community hospital charges reduced the rate of patients with NCDs in district hospitals by 9.4%, while increased the rate in community hospitals by 10.2%. The construction of medical equipment in community medical institutions had reduced the possibility of chronic patients visiting provincial hospitals by 3.5%, and decreased the possibility of visiting district hospitals, while increased the possibility of visiting community hospitals. What’s more, the construction of medical examination services in community hospitals had reduced the probability of patients with NCDs going to provincial hospitals by 2.4%, to district hospitals by 6.8%, and to community hospitals by 3.1%. Notably, the marginal impact of the construction of community privacy protection on NCDs patients in provincial hospitals was 8.8%, and the marginal impact on patients in community hospitals was 5.9%.

**Table 4:** Marginal impact of community medical institution construction content on medical choice of NCDs patients

Item	Provincial hospitals	District hospitals	Community hospitals
Service attitude	-0.003 [-0.050, 0.044]	-0.124^***^ [-0.183, -0.066]	0.127^***^ [0.077, 0.178]
Nursing service	0.037 [-0.011, 0.085]	-0.073^**^ [-0.123, -0.024]	0.036^**^ [0.024, 0.097]
Doctor-patient communication	0.007 [-0.038, 0.052]	-0.004 [-0.051, 0.043]	0.003 [-0.060, 0.053]
Expert consultation	0.009 [-0.025, 0.043]	-0.044^*^ [-0.079, -0.008]	0.035^**^ [0.092, 0.078]
Waiting time	0.012 [-0.048, 0.072]	0.018 [-0.059, 0.043.]	-0.003 [-0.049, 0.041]
Charging standard	-0.008 [-0.042, 0.025]	-0.094^***^ [-0.129, -0.058]	0.102^***^ [0.068, 0.146]
Drug type	0.039 [-0.006, 0.084]	0.018 [-0.032, 0.040]	-0.021 [-0.056, 0.013]
Medical equipment	-0.035^**^ [-0.068, -0.012]	-0.031^**^ [-0.057, -0.015]	0.032^**^ [0.012, 0.064]
Medical examination	-0.024^*^ [-0.057, -0.009]	-0.068^*^ [-0.074, 0.127]	0.031^**^ [0.015, 0.067]
Visit distance	-0.034 [-0.072, 0.004]	-0.011 [0.050, 0.028]	0.045 [-0.003, 0.093]
Privacy protection	-0.088^***^ [-0.135, -0.041]	0.030 [-0.009, 0.068]	0.059^**^ [0.018, 0.098]
Referral	-0.012 [-0.057, 0.034]	-0.004 [-0.057, 0.034]	0.015 [-0.019, 0.050]
Model VCE	OIM		
Number of Obs.	1205		

As shown in Table 5, among the four categories of community medical service construction, external factors had the greatest impact on the medical choice for patients with NCDs, accounting for 42.72%. The second was the medical and nursing quality, with a contribution rate of 23.43%. The third was hardware facilities, with a contribution rate of 22.13%. The cost of diagnosis and treatment ranked last, accounting for 11.71%. From the perspective of specific medical service construction content, the factors that had the greatest impact on the choice of patients with NCDs were privacy protection, medical examination, service attitude, charging standard, and nursing service. The contribution rate of the five construction contents exceeds 80% of the total contribution rate of all the community medical institutions’ construction contents.

**Table 5:** Results of shapley value decomposition

Category	Contribution rate *(%)*	Rank	Item	Contribution rate *(%)*	Rank
Medical and nursing quality	23.43	2	Service attitude	11.71	3
			Nursing service	7.15	5
			Doctor-patient communication	3.54	8
			Expert consultation	1.03	12
Cost of diagnosis and treatment	11.71	4	Waiting time	1.87	10
			Charging standard	9.84	4
Hardware facilities	22.13	3	Drug type	5.14	6
			Medical equipment	4.55	7
			Medical examination	12.44	2
External factors	42.73	1	Visit distance	2.42	9
			Privacy protection	38.91	1
			Referral	1.40	11
Total	100.00	—	—	100.00	—

## Discussion

The current study showed a higher score of the construction content of community medical institutions. And the visiting rate of NCDs patients in community hospitals were at a higher level in southwest China, which was 64.4%. Previous research showed that the proportion rate of visits to primary medical institutions in China was 53.17% in China [21], which was a bit lower than that in this research. A potential explanation is that Southwest China is an underdeveloped region in China, the transportation for residents may not convenient. Especially, provincial and district hospitals are generally located in counties and cities, which increases the possibility of residents choosing community hospitals close to their dwelling. Another explanation is that NCDs patients in underdeveloped areas do not choose medical institutions solely based on the severity of their diseases, but visit within their own economic capabilities [22]. Therefore, the high rate of grassroots visits in underdeveloped areas may not only be due to the good implementation effect of graded diagnosis and treatment, but also more due to economic factors. In fact, previous study has showed that the accessibility of outpatient and inpatient treatment in China presented a distribution pattern of high in the eastern and central China and low in the western [23]. It also further confirmed that there was regional imbalance in Chinese medical resources, and the number of provincial and district hospitals in the underdeveloped areas such as southwest China was insufficient, and the accessibility was low.

In addition to economic factors, age, education level, and the type of medical insurance are also significant factors affecting the medical choices of NCDs patients in underdeveloped areas of Southwest China. Firstly, the medical procedures of district hospitals and provincial hospitals are mostly based on the internet and it is complex and difficult to operate for older NCDs patients. Secondly, it has proved that the income of residents increases with the improvement of education level [24]. NCDs patients with higher education levels tend to choose district or provincial hospitals for treatment due to their higher income and higher medical payment ability [25, 26]. Thirdly, NCDs patients who have basic medical insurance for residents may obtain a larger proportion of reimbursement by choosing to go to community medical institutions [27, 28] thus reducing self-funded medical expenses. While NCDs patients with basic medical insurance for urban employees tend to visit district and provincial hospitals. This may be due to the different levels of medical reimbursement in China. Taking Yunnan Province as an example, the proportion of outpatient reimbursement for residents with employee medical insurance in community hospitals, district hospitals, and provincial hospitals is 60%, 55%, and 50%, respectively, with little difference in reimbursement ratio. While the proportion of outpatient reimbursement for residents with basic resident medical insurance in community hospitals, district hospitals, and provincial hospitals is 50%, 25%, and 25%, respectively [29]. It can be seen that the differentiated reimbursement system of medical insurance has promoted residents to choose primary medical institutions to a certain extent. The differential reimbursement system of basic medical insurance for residents has played a guiding and regulating role in residents’ medical treatment.

Further attempt was made to explore the influencing role of various construction contents of community hospitals to the medical choice of NCDs patients. The results indicate that strengthening service attitude, nursing service, charging standard construction, and enriching the types of commonly used drugs in community hospitals can promote the flow of NCDs patients from district hospitals to community hospitals.

Furthermore, when the construction of medical equipment, medical examinations, and privacy protection are further strengthened in community hospitals, it would also promote the flow of NCDs patients from provincial hospitals to community hospitals. Previous studies have shown that community hospitals have problems with outdated facilities and equipment, limited diagnostic and treatment capabilities of general practitioners, and the medical needs of elderly residents in expert clinics, drug types, and other aspects have not been met [30]. This is consistent with the results of this study. In addition, Another study also showed that community hospitals have the advantage of convenience and speed, but due to the lack of active communication and timely response to consultations with patients by community medical personnel, the advantages of community hospitals have not been fully demonstrated [31]. What’s more, the current research also showed that the construction content of building community medical institutions has a greater diversion effect on district hospitals than on provincial hospitals. In other words, district hospitals would face the dilemma of “sandwich layer”. With the construction of community hospitals and the accuracy self-position, the living space of district hospitals is becoming smaller and smaller. Patients with serious diseases choose provincial hospitals, and patients with minor diseases visit community hospitals. District hospitals are faced with serious survival problems. Moreover, compared with provincial hospitals and district hospitals, the way of queuing and calling in community hospitals is not cumbersome, which is conducive to reducing the waiting time of patients, but the order of medical treatment is also relatively chaotic, so that there are multiple patients in a consulting room at the same time, which is not conducive to the privacy protection of patients.

Therefore, community hospitals should firstly improve the construction of medical order, carry out one-to-one diagnosis and treatment services, and protect the privacy of patients. In addition, the government should subsidize the purchase of medical equipment by community hospitals to improve the medical examination level of community hospitals. In many cases, patients prefer to go to provincial hospitals due to provincial hospitals having advanced medical inspection equipment and the inspection results are highly reliable [32, 33]. Moreover, community hospitals, district hospitals, and provincial hospitals should improve their service attitude. Good service attitude and timely communication with patients would reduce most doctor-patient conflicts [34, 35]. Community hospitals should standardize the charging standards. Meanwhile, the government should strengthen the supervision of the pharmaceutical industry, and put an end to the phenomenon of maliciously raising prices due to the large proportion of reimbursement. Furthermore, district hospitals should actively seek ways out and break the sandwich layer dilemma by transforming to higher level hospitals, strengthening the medical care function and integrating into medical consortiums according to regional characteristics [36, 37]. Finally, patients with NCDs should improve their health literacy [38], make initial visit in community hospitals and cooperate in establishing electronic health records, maintain a good relationship with community doctors in the long term, which is beneficial for doctors to understand the condition, reduce unnecessary examinations, or repeated medication caused by referrals.

Several limitations in this study warrant attention. First, this study was limited to three cities geographical areas in Southwest China. NCDs patients in other Southwest China, especially areas like Guizhou, Guangxi, and Tibet, may have different medical choice. Second, future studies should include investigation on the staff of community hospitals. And thus, add construction content from the perspective of staff in the community hospitals.

## Conclusion

In conclusion, this study explored the medical choice of patients with NCDs from the perspective of the content construction of community hospitals. The study found that the construction content of community hospitals promoted the flow of NCDs patients from district hospitals to community hospitals. Among them, the five contents, privacy protection, medical examination, service attitude, charging standard, and nursing service, have the greatest impact on the medical choice for NCDs patients. This study fills important gaps in previous literature and provides practical experience for the implementation of the new medical reform policy on hierarchical diagnosis and treatment in the underdeveloped areas of southwest China.

## Data Availability

The datasets used and analyzed during the current study are available from the corresponding author on reasonable request.
